# Evaluation of Corneal Biomechanical Properties Modification after Small Incision Lenticule Extraction Using Scheimpflug-Based Noncontact Tonometer

**DOI:** 10.1155/2014/290619

**Published:** 2014-08-31

**Authors:** Leonardo Mastropasqua, Roberta Calienno, Manuela Lanzini, Martina Colasante, Alessandra Mastropasqua, Peter A. Mattei, Mario Nubile

**Affiliations:** ^1^Ophthalmic Clinic, University “G d'Annunzio” of Chieti-Pescara, Via dei Vestini, 66100 Chieti, Italy; ^2^Ophthalmic Clinic, Campus Biomedico, University of Rome, 00185 Rome, Italy

## Abstract

*Purpose.* To quantify the effect of small incision lenticule extraction (SMILE) on the corneal biomechanics using Scheimpflug noncontact tonometer (Corvis ST). *Methods.* Twenty eyes of twenty patients, evaluated as eligible for surgery, with high myopia and/or moderate myopic astigmatism, underwent small incision lenticule extraction (SMILE). All patients underwent Corvis ST preoperatively and postoperatively after 1 week, and 1 and 3 months to observe alterations of corneal biomechanical properties. The main outcome measures were Deformation Amplitude, 1st-AT, and 2nd-AT. The relationship between the amount of stroma removed and the percentage variation of the measured parameters from baseline was evaluated with generalized linear model from each time point. For completeness also intraocular pressure (IOP), central corneal thickness (CCT), and their variations after surgery were evaluated. *Results.* The ratio between the amount of removed refractive error and, respectively, changes of Deformation Amplitude, 1st-AT, and 2nd-AT were significantly modified at the 1st week after surgery (*P* = 0.005; *P* = 0.001; *P* = 0.024). At 1 and 3 months these values did not show statistically significant alterations. Intraocular pressure and central corneal thickness showed statistically significant changes during follow-up. *Conclusions.* No significant modifications in biomechanical properties were observed after SMILE so this procedure could induce only minimal transient alterations of corneal biomechanics.

## 1. Introduction

The structural and reparative properties of the cornea are essential to its function as a resilient, yet transparent, barrier to intraocular injury. Because the cornea is also the scaffold for the major refractive surface of the eye, any mechanical or biological response to injury will also influence optical performance. Consequently, the same mechanisms responsible for preserving ocular integrity can undermine the goals of achieving predictable and stable visual outcomes after keratorefractive surgery [1].

While empirical modifications to algorithms and major advances in laser delivery platforms have improved the statistical predictability of refractive surgery currently most widespread procedures (LASIK, PRK), the ability to anticipate confounding biological responses at the level of the individual patient remains limited. In fact, a predisposition to mechanical instability or abnormal regulation of healing can lead to serious complications such as keratectasia [1]. Previous studies already reported a significant reduction of corneal resistance after LASIK surgery [2–5].

The goal of research in this setting is to improve outcomes and reduce complications by discerning details of the biomechanical and wound healing pathways, identifying measurable predictors of individual responses [1].

In this context the possibility of standardizing the measurement of corneal tissue deformation degree, induced by refractive surgery procedures, would be essential to determine the predictability in the development of complications such as keratectasia and to compare different surgical procedures in terms of biomechanical and tissue stability.

**Figure 1:**
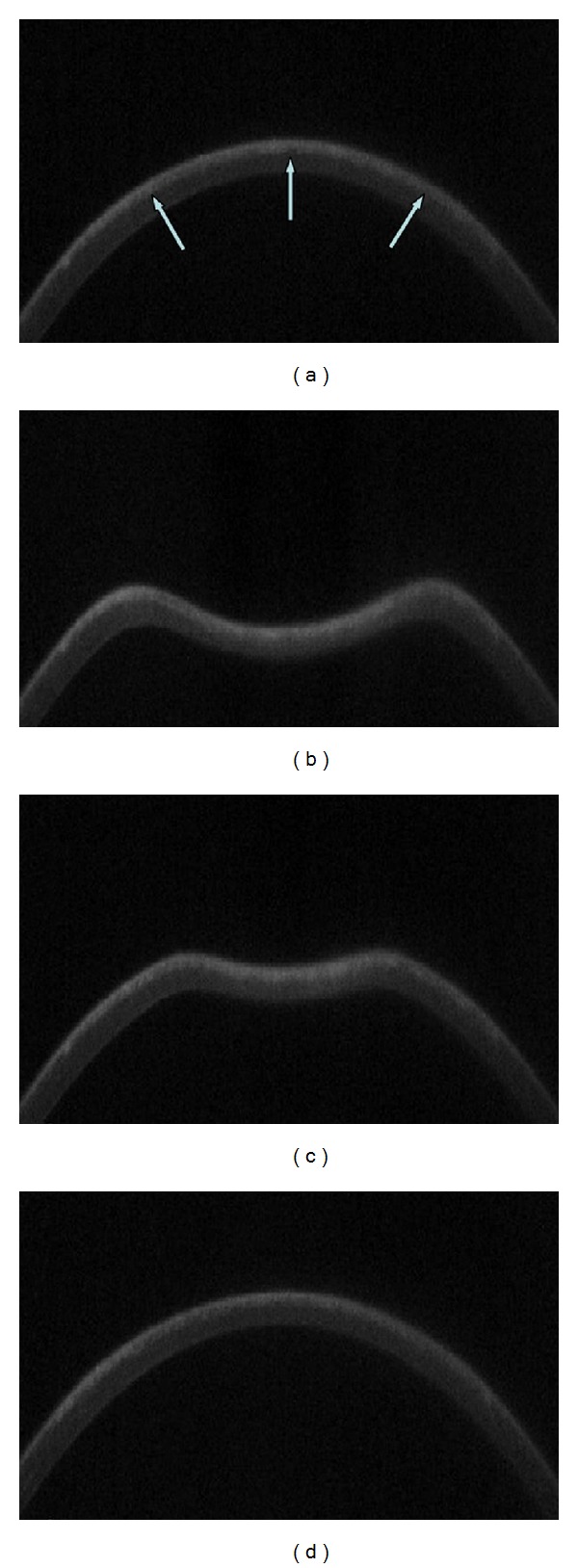
Corvis UHS Scheimpflug camera frames of corneal response to a metered, collimated air pulse: air pulse forces cornea that underwent SMILE procedure (a) inwards through applanation into a concavity phase until it achieves the highest concavity (b). An oscillation period precedes the outgoing or returning phase. The cornea undergoes a second applanation (c) before achieving its natural shape with possible oscillation (d). White arrows indicate femtosecond laser cutting surface interface after SMILE procedure.

The Oculus Corvis ST (Oculus Optikgeräte, OCULUS, Wetzlar, Germany) is a noncontact High-Speed (UHS ST) tonometer, supported by Scheimpflug Technology, designed to obtain in vivo measurements of corneal biomechanical properties; this device allows monitoring corneal deformation response to a symmetrically metered air pulse (Figure 1).

**Figure 2:**
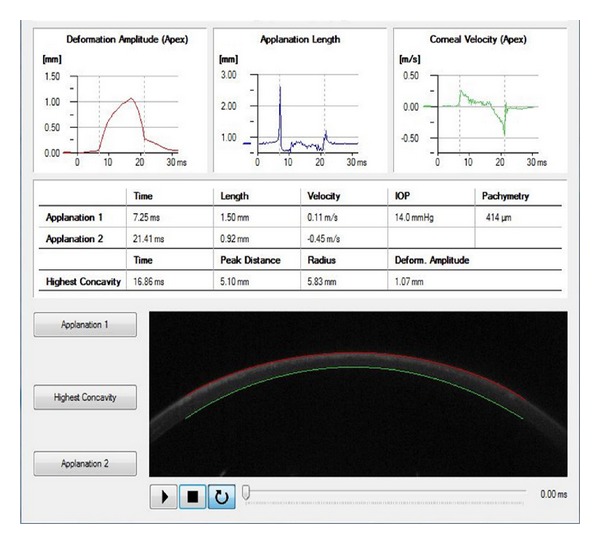
Measurements obtained by Corvis immediately upon air impulse after SMILE procedures. Real-time informations recorded after SMILE: corneal highest concavity, IOP, pachymetry, and first and second time applanation. A high-speed Scheimpflug camera recorded the cornea movements and then displayed them on the control panel in an ultraslow motion (not shown).

The device depicts the time required to applanate the cornea with the air puff, and the time of the first inward applanation is directly proportional to the IOP, which ranges from 1 mm Hg to 60 mm Hg. IOP and CCT are obtained during one measurement process. Additional Corvis ST parameters are measured in time in milliseconds, length in millimetres, and velocity in metres/second of the first (air puff flattens cornea) and second (interruption of air puff results in “reformation” of cornea) corneal applanation; furthermore, peak distance, radius, and deformation amplitude in millimetres of the highest corneal concavity during the measurement process are measured [6] (Figure 2).

Different previous studies demonstrated that corneal parameters measured by Corvis ST are repeatable and reproducible [6–9]; in particular Hon and Lam recently defined the central corneal thickness (CCT) as the most repeatable corneal parameter measured by Corvis and the deformation amplitude (DA) as an indicator of corneal biomechanical properties, followed by the first applanation time (1st-AT) [8].

With the introduction of the VisuMax femtosecond laser (Carl ZeissMeditec AG, Jena, Germany) in 2006 [10, 11], keratorefractive surgery was revolutionized and intrastromal keratomileusis was reinvented in the shape of refractive lenticule extraction [12]. A new procedure, small-incision lenticule extraction (SMILE), was developed, totally without excimer laser support.

**Figure 3:**
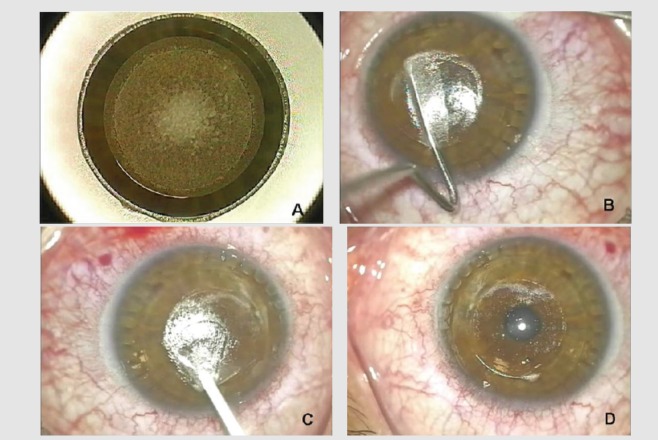
Femtosecond laser SMILE procedure: a stromal lenticule, with characteristics defined on the basis of the refractive defect of the patient, is cut within the corneal stroma by the femtosecond laser (a). Afterwards, only a small incision is made to allow access to dissect (b) and manually remove the lenticule (c-d).

During SMILE procedure, first a stromal lenticule, with characteristics defined on the basis of the refractive defect of the patient, is cut within the corneal stroma by the femtosecond laser, using ultrafast [13–18] pulses to create photo disruption. Afterwards, a surface cut is made to allow access to dissect and manually remove the lenticule [11]. In SMILE, only a small incision is made without the creation of a flap, minimizing trauma to the corneal surface if compared with other surgical procedures (PRK or LASIK) [19–22] (Figure 3).

In a recent paper, Hassan and coll. carried out a comparison of the corneal biomechanical parameters variation between PRK and LASIK using Corvis; they observed that most of these biomechanical parameters remained unchanged after one month of LASIK and PRK compared to the preoperative data [23].

Also a recent study of Reinstein et al., developing a mathematical model to estimate the relative differences in postoperative corneal stromal tensile strength following PRK, LASIK, and SMILE, defined that the postoperative stromal tensile strength is higher after SMILE if compared with other procedures, as expected given that the strongest anterior lamellae remain intact in SMILE [24].

However, little is known regarding the biomechanical effects of SMILE. Provided that all types of keratorefractive surgery induce a variable degree of corneal deformation with consequences on tissue stability, it is interesting to assess the possible biomechanical alterations induced by SMILE procedure.

So the aim of this study was to quantify the effect of SMILE on the corneal biomechanical properties, for the first time, by means of Corvis ST.

## 2. Materials and Methods

This prospective, nonrandomized, and comparative clinical trial comprised 20 eyes of 20 patients (age from 25 to 43 years, DS 34 ± 12), scheduled for refractive surgery at the Ophthalmic Clinic of the “SS. Annunziata” Hospital of Chieti (Italy) for myopic and/or astigmatic correction between May 2010 and October 2012, that underwent SMILE. The protocol adhered to the tenets of the Declaration of Helsinki and received approval from an institution review board. Informed consent was obtained from all participants and possible consequences of taking part were explained.

Inclusion criteria were a moderate to high myopia, stability for at least 1 year, a corrected distance visual acuity (CDVA) of 20/25 or better, spherical equivalent refraction from −3.00 to −7.00 diopters (D), and a refractive astigmatism from 0.50 to 1.50 D.

A central corneal thickness (CCT) less than 480 *μ*m, a calculated postoperative residual stromal bed of less than 250 *μ*m, a presence of keratoconus, pregnancy, or breastfeeding, and all other ocular pathological conditions meant exclusion from surgery.

Patients underwent eye examination including objective and manifest visual acuity, intraocular pressure (Canon tonometer, TX10, NY, USA), pupil size (Sirius, CSO, Firenze Italy), keratometric measurements, slit-lamp examination, and fundoscopy (Slit lamp BM900, Haag-Streit, Koeniz Switzerland). Regular topographic patterns of both the corneal front and back and normal CCT were confirmed with a Pentacam-HR Scheimpflug camera (Oculus, Germany). This included the usage of the Pentacam Ambrósio/Belin module to exclude also a subclinical keratoconus. We decided to consider the most repeatable indicators of corneal biomechanical properties measured by Corvis ST and already validated from scientific literature [8]: the deformation amplitude (DA) and the first applanation time (1st-AT). We also voluntarily considered, for study completeness, the second applanation time (2nd-AT). Also IOP and CCT values were evaluated before surgery and at every step of follow-up in order to identify modifications of these parameters induced by SMILE.

All patients before surgery underwent Corvis ST measurement preoperatively to evaluate DA that represents the maximum amplitude at the corneal apex (highest concavity), 1st-AT, that is, the time from starting until the first corneal applanation, and 2nd-AT, that is, time from starting until the second applanation, according to the mode previously described in literature [24] and described below.

The patient can be comfortably positioned due to the proper placement of the chin and forehead and is asked to focus at the central red light emitting diode (LED).

The UHS Scheimpflug camera takes over 4,300 frames per second in order to monitor corneal response to a metered, collimated air pulse with symmetrical fixed profile and a fixed maximal internal pump pressure of 25 kPa.

This imaging system permits the dynamic inspection of the actual deformation process during noncontact tonometry. The recording starts with the cornea at the natural convex shape. The air pulse forces the cornea inwards (i.e., the ingoing phase) through applanation (i.e., the first or ingoing applanation) into a concavity phase until it achieves the highest concavity (HC). An oscillation period precedes the outgoing or returning phase. The cornea undergoes a second applanation before achieving its natural shape with possible oscillation (Figure 1). The timing and corresponding pressure of the air pulse at the first and second applanation and at the HC moments are identified. IOP is calculated based on the timing of the first applanation event. The deformation amplitude (DA) is measured as the highest displacement of the apex in the HC moment image. The radius of curvature at the HC is recorded. The lowest value is displayed. The Corvis ST measurements were repeated on the patients three times subsequently on the same patient by the same operator (Roberta Calienno)

Two surgeons performed all surgical procedures (Leonardo Mastropasqua and Mario Nubile).

Visumax (Carl Zeiss Meditec, Jena, Germany) femtosecond laser platform was used to realize SMILE procedure. The procedures were performed as previously described and illustrated in detail [22, 25, 26].

Briefly, laser cut energy index range was approximately from 125 to 170 mJ and spot spacing ranged from 2.5 to 4.5 *μ*m. Then a 40° to 60° incision located at the 12-o'clock position was created to allow the lenticule extraction. Lenticule diameter (optical zone) was 6.0 to 6.5 mm, and the cap diameter was 7.3 mm. Intended cap thickness was 110 to 120 *μ*m.

After surgery, all patients received one drop of Netilmicin 0,3% (Nettacin, SIFI, Catania, Italy) and one drop of dexamethasone phosphate 0,15% (Etacortilen, SIFI, Catania, Italy); then a soft contact lens was applied.

The postoperative regimen included the same eye drops four times a day for 1 week, followed by two times a day for 1 week and lubricating drops four times a day. On the first day after surgery, the soft contact lens was removed; then visual acuity was measured and slit-lamp examination was performed. On the first week and 1 month and 3 months after surgery, patients returned for a follow-up examination with measurement of the same parameters and Corvis ST parameters.

### 2.1. Statistical Analysis

Parameters were summarized as mean and standard deviation of the percentage difference from baseline values. The presence of statistically significant differences in the percentage variation from baseline for each of the three variables was evaluated with a paired* t*-test. The relationship between the amount of stroma removed and the percentage variation of each of the measured parameters from baseline was evaluated with linear regression for each time point.

Statistical analysis was performed using SPSS 20.0 (IBM, Armonk, NY). Statistical significance was assigned at *P* ≤ 0.05.

In addition IOP and CCT values were considered to identify if a statistically significant difference was present between preoperative time and all follow-up times with an ordinary one way ANOVA test.

## 3. Results

The surgical procedure was successfully completed in all patients, no complications occurred, and no patients were lost to follow-up. Mean correction was 5.5 ± 1.2 D (range 3.5 to 7 D) at each of the three follow-ups.

Table 1 shows the percentage variation from baseline values of the three parameters expressed as mean and standard deviation (S.D.) for each of the three time points that were compared using paired* t*-tests. The differences between seven days and 30 and 90 days were statistically significant for all three parameters while those between 30 and 90 days were not.

**Figure 4:**
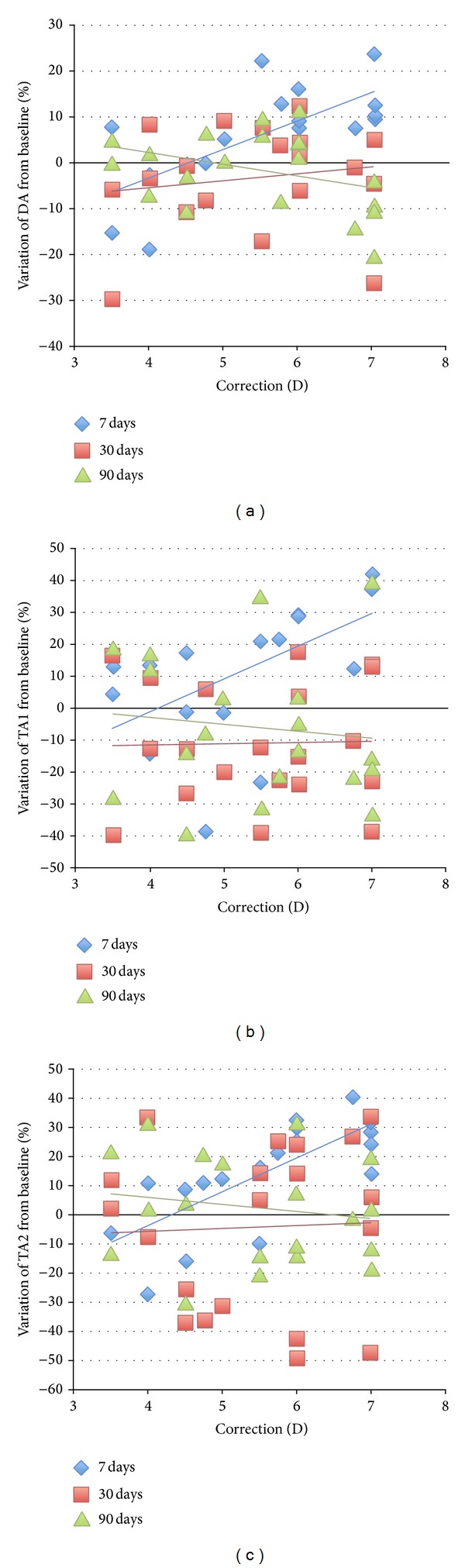
The figure presents a scatterplot of the percentage variation from baseline values of DA (a), TA1 (b), and TA2 (c) for each subject against the correction performed with SMILE.

Figure 4 presents a scatterplot of the percentage variation from baseline values of DA (a), TA1 (b), and TA2 (c) for each subject against the correction performed with SMILE. These relationships were evaluated with linear regression analysis that showed a statistically significant relationship was present only at 7 days postoperatively. The coefficient of determination (*r*2) was 0.502 for D (*P* < 0.001), 0.347 for TA1 (*P* = 0.004), and 0.583 for TA2 (*P* < 0.001) at seven days postoperatively. The regression lines also demonstrated that there was a pattern for less structural integrity with progressively higher corrections at seven days postoperatively but not at the later follow-ups.

The variation from preoperative values of the IOP and CCT and all time of follow-up, compared using ordinary one way ANOVA, showed a statistically significant difference (reduction) (*P* = 0.0082 and *P* = 0.0084, resp.).

## 4. Discussion

Corneal biomechanical properties involve, besides elasticity and viscosity, thickness and hydration, mostly dominated by the stroma that constitutes 90% of the total corneal thickness and determines mechanical response of the cornea to injury [27].

Despite the recent progress and the technological advances that are obtained in refractive surgery, particularly in improved understanding of the refractive errors basic science and the biomechanics of corneal wound healing, currently complications still happen [28].

In fact, the unpredictable nature of corneal wound healing and the biomechanical response to surgery can lead to postoperative refractive surprises, discrepancies between attempted and achieved visual outcomes, and biomechanical and wound healing problems with particular importance for keratectasia [28]. During LASIK, PRK, or any other procedure involving central ablation, an immediate circumferential severing of corneal lamellae is produced. In simple elastic shell models, this results in a forward herniation that would result in corneal steepening and thickening [29]. It is already known that LASIK flap creation may induce astigmatism and higher order aberrations [30, 31] because the flap itself is subject to shape changes induced by the circumferential keratotomy of flap creation.

Furthermore, the risk of developing post-LASIK ectasia increases in patients with preexisting keratoconus, deep flaps, high myopia, deep laser ablation [32], a corneal thickness lower than 500 *μ*m, and residual stromal bed thickness lower than 250 *μ*m.

Since refractive surgery decreases collagen tension, by disrupting cornea biomechanics, and may lead to corneal ectasia that favors decreased visual acuity [33], determining risk factors which lead corneal ectasia and understanding what kind of refractive surgical procedure can reduce ectasia incidence are indispensable.

Femtosecond laser seems to imply biomechanical advantages in LASIK flap creation [18].

SMILE procedure theoretically may have biomechanical benefits over LASIK because it does not involve the creation of a flap and leaves the stroma over the lenticule untouched. However, there are not many published studies regarding the biomechanical effects of SMILE.

Recently Agca et al. performed an analysis of corneal biomechanical properties (corneal hysteresis as CH and corneal resistance factor as CRF) after SMILE, comparing these values with the same ones obtained after femto-LASIK procedure with the support of the Ocular Response Analyzer (ORA; Reichert Inc., Buffalo, NY, USA) [34].

They concluded that there were no differences between the two compared procedures in terms of biomechanical properties and that CH and CRF decreased after SMILE.

They also hypothesized that, although not statistically significant, differences in postoperative CH and CRF values between the femto-LASIK and SMILE groups were found; this did not mean that the corneas in both groups were biomechanically similar to each other after these surgical procedures because CH and CRF values only reflected some clinically significant aspects of corneal biomechanical structure and the absence of difference did not mean that the corneas in both groups are biomechanically similar [34].

In order to investigate these critical points, our study aims to assess changes induced on corneal biomechanical parameters after SMILE to understand the impact of this new surgical procedure on corneal biomechanical stability.

We decided to consider the most currently validated CORVIS parameters by the scientific literature: DA and applanation times [8]. In addition, IOP and CCT were considered because it is already known by scientific literature that these parameters are fundamental in corneal deformation response evaluation and could influence the CORVIS biomechanical values [35].

In particular we identify a statistically significant difference for both parameters between the preoperative period and all the follow-up period as might be expected after SMILE treatment.

Such significance certainly makes our biomechanical results more valid and reliable.

Moreover, as described in the results section, DA and 1st and 2nd applanation times were increased 7 days after surgery. This is easily understood since the procedure SMILE, causing the removal of a corneal tissue lenticule, reduces the stiffness and the structural compactness of the cornea and consequently involves an increase of applanation times.

In fact, as it is guessed, thinner and therefore less rigid corneas have more applanation time because when a load force is applied over it, the corresponding response force is reduced as it is directly related to the decreased tissue stiffness.

Consequently also the amplitude of deformation will increase by increasing the corneal deformability. However at the other follow-up controls (30 and 90 days) all the three parameters presented no statistically significant modifications.

So, based on our results, a substantial modification of corneal biomechanics occurs only in the very first follow-up time after SMILE (7 days).

This is certainly related to the lenticule removal and the subsequent rebuilding of a new biomechanical balance dominated by different tensile forces related to the residual stromal bed. Otherwise, these differences from baseline were evident, and a statistically significant relationship was present, only at 7 days postoperatively but not between 30 and 90 days; this probably means that this new biomechanical balance is relatively quickly determined, and this despite the removed lenticule thickness that is directly proportional to the corrected refractive errors.

This would mean that corneal biomechanical stability is only relatively and temporarily modified after SMILE, when stromal lenticule removal, graven by femtosecond laser, happens. So probably tensile forces that allow and encourage corneal stability are altered only minimally after SMILE procedure.

On the basis of the most recent scientific literature that defined SMILE as a minimally invasive and inflammatory surgical procedure for corneal tissue if compared to the other refractive surgery procedures [36], we could also hypothesize that even a low level of induced inflammation may perhaps encourage a more rapid reestablishment of a stable biomechanical corneal balance.

However the study presents limits of a reduced sample size and the use of Corvis ST, that is, an innovative imaging system that allows obtaining in vivo biomechanical information and avoids the limitations of previous in vivo and in vitro techniques but is only recently emerging as a clinical instrument used to investigate in vivo biomechanical properties of the cornea.

Probably other prospective studies supported by the use of this new technology are needed to confirm our data and to imply the corneal biomechanical properties knowledge. Furthermore, as already reported in literature [33], the average appearance timing of corneal ectasia is about 12–60 months after LASIK; so it will be interesting to conduct a more extensive follow-up (up to 3 months) in order to consider possible late changes of corneal biomechanics after SMILE.

Finally, a comparison on the corneal biomechanical changes after SMILE among patients who presented a variability of myopia and astigmatism degree (from minimum to high) will certainly be an additional element of assessment (our patients presented only high level of myopia and/or moderate myopic astigmatism).

In conclusion, from these results we could define SMILE as a procedure that determines only minimum alterations of corneal biomechanics but we need to overcome our limitations by broadening and deepening the study in order to well define the benefits of this new and increasingly diffuse refractive surgery procedure.

## Figures and Tables

**Table 1:** Percentage variation from baseline values of the three parameters measured (millisecond (ms) in applanation times and millimeter (mm) for deformation amplitude) expressed as mean and standard deviation (S.D.) for each of the three time points. The three time points were compared using a two-tailed paired *t*-test.

	*N*	Mean ± S.D	Paired *t*-test	
			*P* (versus 7 days)	*P* (versus 30 days)
DA (mm)				
7 Days	20	60.0 ± 10.4	0.005	
30 Days	20	−3.6 ± 11.2	0.021	
90 Days	20	−1.7 ± 8.4		0.576
TA1 (ms)				
7 Days	20	14.8 ± 21.7	0.001	
30 Days	20	−10.7 ± 18.9	0.014	
90 Days	20	−6.0 ± 21.9		0.263
TA2 (ms)				
7 Days	20	13.2 ± 18.1	0.024	
30 Days	20	−4.0 ± 28.3	0.049	
90 Days	20	2.4 ± 18.6		0.385
